# Impact of the TCO Microstructure on the Electronic Properties of Carbazole-Based Self-Assembled Monolayers

**DOI:** 10.1021/acsmaterialslett.3c01166

**Published:** 2023-12-26

**Authors:** Suzana Kralj, Pia Dally, Pantelis Bampoulis, Badri Vishal, Stefaan De Wolf, Monica Morales-Masis

**Affiliations:** † MESA+ Institute for Nanotechnology, University of Twente, Enschede 7500 AE, The Netherlands; ‡ Physics of Interfaces and Nanomaterials, MESA+ Institute for Nanotechnology, University of Twente, Enschede 7500 AE, The Netherlands; § KAUST Solar Center (KSC), Physical Sciences and Engineering Division (PSE), King Abdullah University of Science and Technology (KAUST), Thuwal 23955-6900, Kingdom of Saudi Arabia

## Abstract

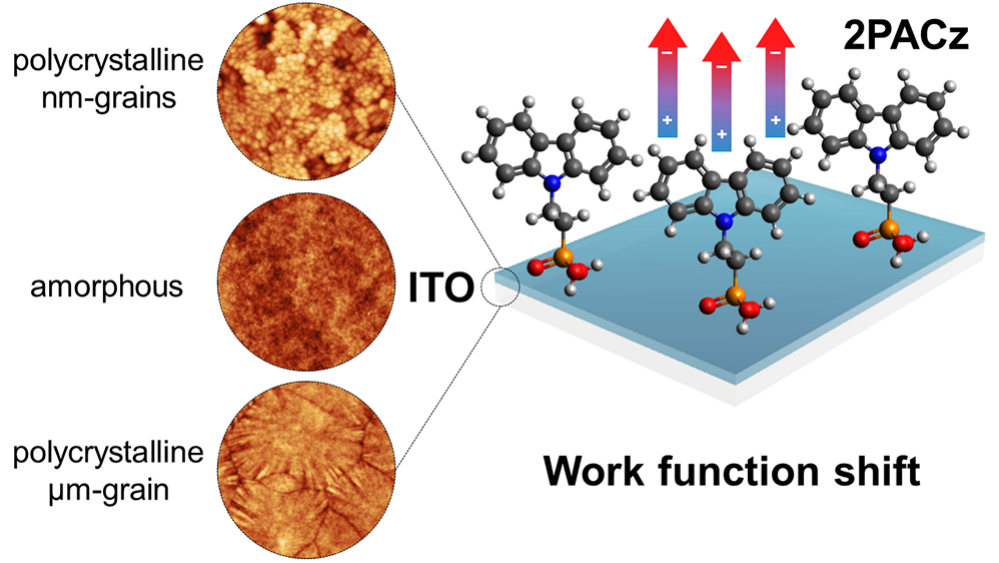

Carbazole-based self-assembled monolayers (PACz-SAMs), anchored via their phosphonic acid group on a transparent conductive oxide (TCO), have demonstrated excellent performance as hole-selective layers in perovskite/silicon tandem solar cells. Yet, whereas different PACz-SAMs have been explored, the role of the TCO, and specifically its microstructure, on the hole transport properties of the TCO/PACz-SAMs stack has been largely overlooked. Here, we demonstrate that the TCO microstructure directly impacts the work function (WF) shift after SAM anchoring and is responsible for WF variations at the micro/nanoscale. Specifically, we studied Sn-doped In_2_O_3_ (ITO) substrates with amorphous and polycrystalline (featuring either nanoscale- or microscale-sized grains) microstructures before and after 2PACz-SAMs and NiO_*x*_/2PACz-SAMs anchoring. With this, we established a direct correlation between the ITO crystal grain orientation and 2PACz-SAMs local potential distribution, i.e., the WF. Importantly, these variations vanish for amorphous oxides (either in the form of amorphous ITO or when adding an amorphous NiO_*x*_ buffer layer), where a homogeneous surface potential distribution is found. These findings highlight the importance of TCO microstructure tuning, to enable both high mobility and broadband transparent electrodes while ensuring uniform WF distribution upon application of hole transport SAMs, both critical for enhanced device performance.

Transparent conductive oxides (TCOs) are omnipresent in a range of high-efficiency optoelectronic devices, including perovskite solar cells (PSCs), both in their single-junction and tandem implementations.^1,2^ Among the various available TCOs, indium tin oxide (ITO) remains a common choice as a transparent electrode for optoelectronic applications as it is well established and scalable, and commercial glass/ITO substrates are readily available. However, further progress in PSC performance could benefit from additional TCO optimization, for instance, by enhancing the transparency/conductivity trade-off. Moreover, with the rise of monolithic perovskite/perovskite and perovskite/silicon tandem solar cells, TCOs are also often used as an interband recombination junction^3^ deposited onto the bottom cell (i.e., a perovskite or silicon cell), connecting the subcells in series. Both in the single-junction and tandem cases for inverted *p-i-n* devices, the hole transport layer (HTL) is deposited onto the TCO, followed by the perovskite absorber and the electron selective contact stack. Depending on the TCO deposition method and process conditions, different microstructures can be achieved which may also influence the optoelectronic properties. For instance, amorphous TCOs generally feature a narrower band gap as compared to polycrystalline TCOs due to their distorted absorption edge.^4,5^ The electron mobility in TCOs can also be influenced by the microstructure, with typically high mobilities achieved for polycrystalline TCOs with large grains.^5−9^

For the case of HTLs for inverted PSCs, self-assembled monolayers (SAMs), such as carbazole-based with a phosphonic acid anchoring group (PACz-SAMs), have attracted much attention in recent years.^10−12^ These SAMs shift the work function of the TCO substrate to higher values due to the interfacial dipole they introduce, enhancing the hole selectivity of the contact.^12−14^ So far, the TCO/PACz-SAMs stack has gained particular attention as a hole selective contact for inverted perovskite,^15−21^ organic,^22,23^ perovskite/organic,^24^ perovskite/perovskite,^25−27^ and recent record perovskite/silicon tandem solar cells^28,29^ where often they have been found to result in superior passivation of the HTL/photoabsorber interface, a fast hole extraction rate, and minimal parasitic absorption.^30^ On the downside, the presence of imperfections in PACz-SAMs coatings on polycrystalline TCO is frequently reported,^30−33^ preventing optimal device performance and stability. So far, addressing this challenge has primarily involved either blending SAM molecules of varying sizes to ensure high packing density^26,34−37^ or anchoring the PACz-SAMs to a hole-selective metal-oxide buffer layer, such as nickel oxide (NiO_*x*_).^32,38−40^ The effect of the TCO substrate surface properties, such as roughness, morphology, and composition, on the quality of phosphonic acid-based SAMs has been studied before.^41−43^ However, to the best of our knowledge, no comprehensive investigation into the correlation between the microstructure of TCOs, specifically the grain orientation, and the hole transporting properties of TCO/PACz-SAMs stacks has been reported. To elucidate this, herein, we study the effect of 2PACz, [2-(9H-carbazol-9-yl)ethyl]phosphonic acid (Figure S1), a commonly used SAM hole selective contact, on the WF shifts of various ITO substrates. Furthermore, we investigate the potential distribution and its link to the WF along the surface of distinct types of ITO substrates with different microstructures. In this work, we focus on ITO as a model system, but the conclusions are valid for other TCOs. Specifically, we studied ITO substrates with comparable sheet resistance but distinct microstructures, namely: commercial ITO, featuring a polycrystalline microstructure with small (nm-scale) grains, and pulsed laser deposited (PLD) ITO, either amorphous or polycrystalline with large (μm-scale) grains. Moreover, the effect of introducing a sputtered NiO_*x*_ layer between the different ITO electrodes and 2PACz-SAMs was analyzed. The potential distribution was mapped by using Kelvin probe force microscopy (KPFM), while the grain orientation of the ITO for the same area was measured by electron backscatter diffraction (EBSD) analysis. Ultraviolet photoelectron spectroscopy (UPS) was used to determine absolute WF values and verify the values determined by KPFM. Based on these experiments, we demonstrate how the ITO crystalline grain orientation and grain size influence the potential distribution in the ITO/2PACz-SAMs electrodes, compare the WF values achieved for the distinct ITOs, and discuss the role of a NiO_*x*_ buffer layer on achieving a uniform potential distribution for hole extraction. These insights are invaluable for advancing solar cells and other optoelectronic device designs, ultimately leading to improvements in the efficiency, stability, and reliability.

## Experimental Section

**Figure 1:**
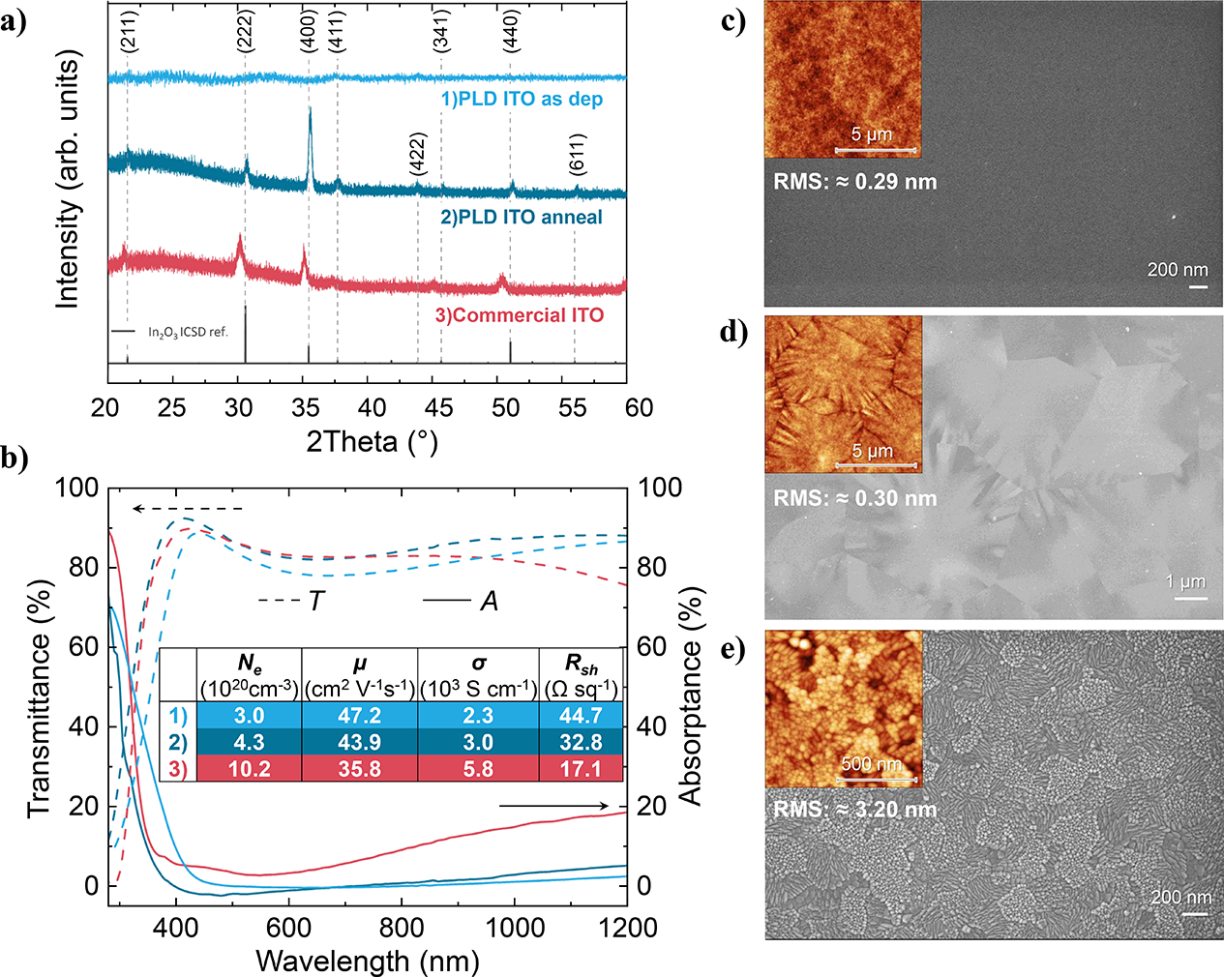
(a) X-ray diffractogram; (b) Optical and electrical properties of studied ITOs. SEM top-view images with an AFM topography inset and RMS values for (c) PLD ITO as deposited; (d) PLD ITO annealed; and (e) Commercial ITO. The thickness of all films is ≈100 nm.

100 nm Sn-doped In_2_O_3_ (10/90 wt % SnO_2_/In_2_O_3_) thin-films with sheet resistance (*R*_*sh*_) below 50 Ω/sq, representing typical TCO device requirements, with three different microstructures were selected for this study. The ITO films were deposited on glass substrates by PLD at room temperature. As-deposited ITO films were found to be amorphous; subsequent annealing for 20 min at 450 °C (Figure S2) resulted in a polycrystalline structure, as confirmed by XRD (Figure 1a, Table S1). Top-view SEM and AFM scans (Figure 1c-d) show a flat, featureless surface for the amorphous ITO (RMS of 0.29 nm) and large micrometer-sized grains for the annealed ITO films (RMS of 0.30 nm). This observed change in crystallinity and microstructure is due to a solid phase crystallization, as previously reported for sputtered In-based TCOs.^7,8^ In the process of physical vapor deposition of In-based TCOs at room temperature (by either PLD or sputtering), nanocrystals are generated within an amorphous matrix. These nanocrystals act as nucleation sites, facilitating the growth of grains during a subsequent annealing step.^7^ Commercially available ITO substrates (Ossila Ltd.), featuring a polycrystalline structure and nanoscale grains microstructure (RMS of 3.20 nm), as depicted in Figure 1a and e, respectively, were used to compare the influence of the grain size in the WF distribution. Figure S3 showcases the comparison of three commercially available ITO substrates from different suppliers. The data indicate that while the optical and electrical properties are within the same range for the three commercial ITOs, there is a slight difference in the surface morphology and roughness. Mainly, the thicker ITO (from supplier 3) shows slight variations in grain distribution and height, which could affect the quality of the SAMs coating.^41,42^ This minor differences in roughness could lead to lab-to-lab reproducibility of the quality of SAMs. Nevertheless, for the purposes of this study and based on the proximity on the ITO properties, we have selected one representative commercial ITO (supplier 1).

All studied ITO films demonstrate >80% transmittance in the wavelength range of 350–750 nm (Figure 1b). For wavelengths above 750 nm, the commercial ITO samples exhibited a larger absorption (>15%) as compared to PLD ITO. This can be explained by free carrier absorption as the concentration of free carriers, *N*_*e*_, is significantly higher (10 × 10^20^ cm^–3^) for commercial ITO films as compared to PLD films (up to 4.5 × 10^20^ cm^–3^). The electrical properties of the ITO films determined from Hall effect measurements in the van der Pauw configuration are summarized in the inset of Figure 1b. Additionally, an increase in the optical band gap for PLD ITO annealed films from ∼3.4 eV for as-deposited ITO to ∼3.7 eV for annealed ITO was estimated from the Tauc plot in Figure S4.

For conciseness, as-deposited ITO films will further be referred to as “*a-ITO*”, annealed ITO polycrystalline films with large (μm-sized) grains will further be referred to as “*poly-ITO-μm-grain*”, and commercial ITO polycrystalline films with small (nm-sized) grains will further be referred to as “*poly-ITO-nm-grain”*.

**Figure 2:**
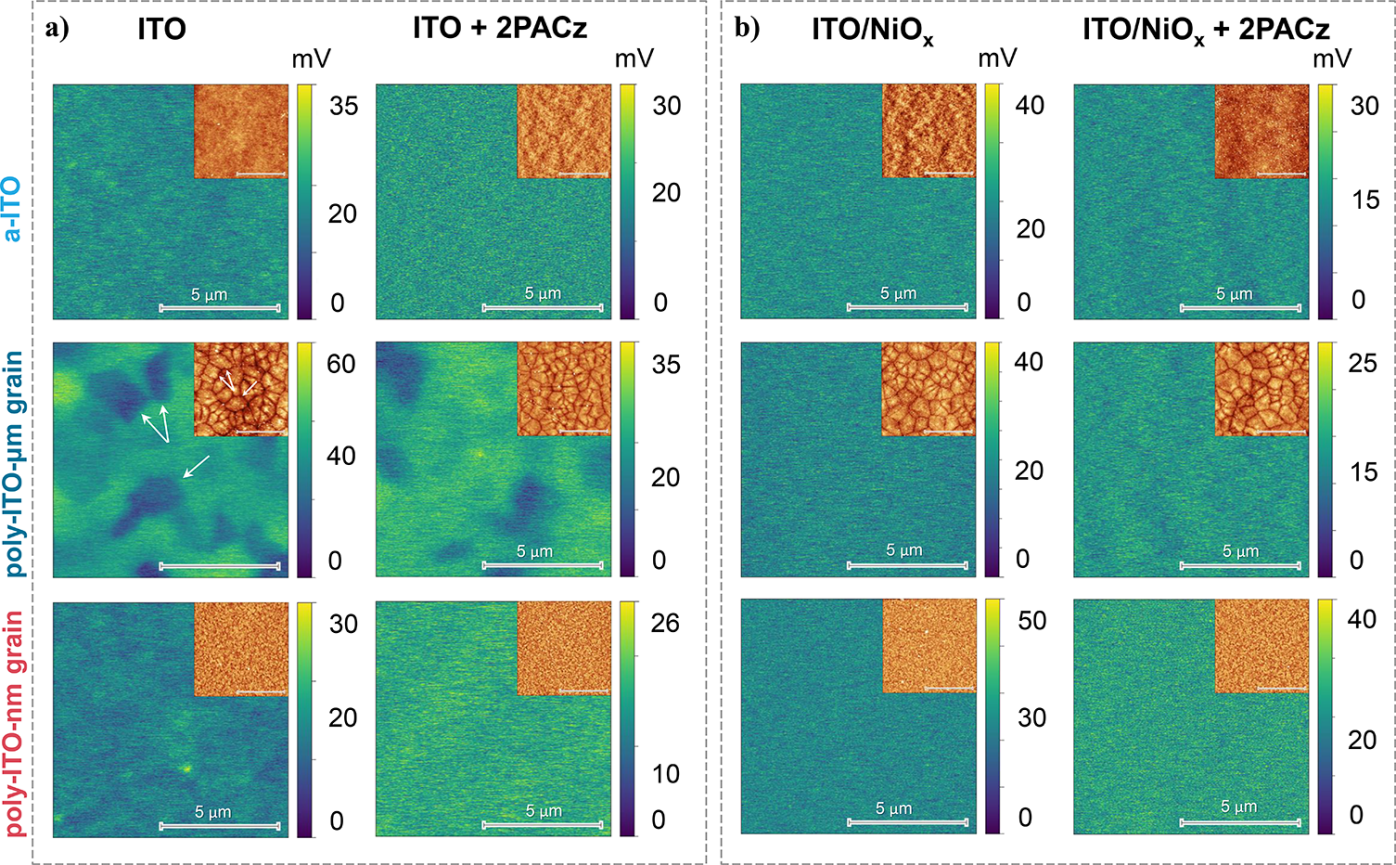
KPFM mapping for the stacks: (a) ITO with and without 2PACz-SAMs; (b) ITO/NiO_*x*_ with and without 2PACz-SAMs (scanning area 10 × 10 μm^2^, larger image: CPD mapping; inset: topography. Note: To ensure a precise depiction of CPD values for each sample, the color scale bar has been adjusted to accommodate the observed deviations. The scale bar of all images, including insets, is equivalent to 5 μm.).

To gain insight into the surface potential distribution of the ITO films with distinct microstructural properties, KPFM was performed, which directly maps the contact potential difference (CPD) between a conducting AFM tip and the sample (Figure S5). Here, a relatively large area of 10 × 10 μm^2^ was scanned. The resulting CPD maps and the corresponding topography image (inset) are shown in the left column of Figure 2a. In the case of *a-ITO* and *poly-ITO-nm-grains*, a relatively consistent surface potential across the scanned film area is measured, indicating an overall homogeneous WF distribution. However, in the case of *poly-ITO-μm-grain* films, the CPD is not uniform across the scanned area, as observed by the lighter and darker domains in Figure 2a, representing areas of higher and lower WF values, respectively. The domains match the corresponding large grains in the topography inset, as marked with white arrows.

Subsequently, 2PACz-SAMs were deposited onto the three ITO films described above (details in the SI). The right column in Figure 2a displays the CPD maps, accompanied by insets of topographic AFM images of the ITO/2PACz-SAMs substrates. Generally, the topography remained virtually unaltered with the introduction of 2PACz-SAMs, but a systematic overall reduction in CPD confirms the presence of 2PACz-SAMs on the surface and implies an increase in the WF. Notably, in the case of the *poly-ITO-μm-grain* films, the presence of domains with distinct CPD values remains even after the 2PACz-SAMs application. For the poly-ITO-μm grain films, a narrowing of the CPD-distribution is observed. We hypothesized that this can be a combined effect of the UV–O_3_ plasma treatment before the application of SAMs which ensures an oxygen terminated surface (and a hydroxyl-rich surface upon exposure to ambient air) and by a quenching effect, which is related to the interaction dynamics between the tip, the surface, and the 2PACz molecules as explained in the SI and Figure S6.

NiO_*x*_ is a p-type, high work function metal oxide with good transparency and has been demonstrated as an effective hole transport layer in inverted PSCs, deposited through a variety of methods, including sputtering.^44,45^ However, it is widely reported that its direct contact with the perovskite absorber leads to a defective interface, leading to the development of several NiO_*x*_ surface passivation approaches, including the use of PACz-SAMs.^32,46,47^ While similar performances have been reported for PSCs with ITO/2PACz-SAMs and ITO/NiO_*x*_/2PACz-SAMs, it has been proposed^40,47^ that the use of a thin NiO_*x*_ buffer layer deposited on top of ITO helps to homogenize morphological and energetical differences on the ITO substrates, enabling higher reproducibility in devices for the ITO/NiO_*x*_/2PACz-SAMs stack as compared to the ITO/2PACz-SAMs counterpart.^32,39^ It is hypothesized that the presence of a NiO_*x*_ buffer layer offers a dual function. First, it acts as a barrier, preventing a direct contact between the perovskite layer and the TCO in the case of pinhole formation within the PACz-SAM layer. Simultaneously, in the event of pinhole formation, the NiO_*x*_ hole-selective nature enables efficient charge carrier extraction, mitigating potential adverse effects. Furthermore, it enhances substrate surface properties, improving PACz-SAMs coverage.^32,38,40,42^ Most of the reported NiO_*x*_ hole transport layers are either amorphous or nanocrystalline, with randomly oriented nanometer-sized grains. We suggest that this amorphous or nanocrystalline microstructure is also beneficial for homogenizing the surface roughness and the surface potential.

To confirm this, here, we sputtered an amorphous NiO_*x*_ layer (14 nm) onto the studied ITOs. Figure S7 displays a featureless X-ray diffraction pattern, confirming the formation of an amorphous NiO_*x*_ film. For elaborate characterizations and properties of the NiO_*x*_ layer, we refer the reader to ref (44). We note that NiO_*x*_ subsequently underwent a treatment with a potassium chloride (KCl) solution to passivate its surface defects.^48^ Topography and surface potential images for the resulting ITO/NiO_*x*_ stack with and without 2PACz-SAMs are shown in Figure 2b. The left column illustrates CPD mappings and topography insets for the ITO/NiO_*x*_ configuration. AFM images reveal that the NiO_*x*_ layer follows the topological features of the distinct ITO substrates. However, the presence of NiO_*x*_ on the surfaces of the ITO films clearly reduces the variations in the CPD values along the surfaces of all ITOs. Consequently, a uniform surface potential distribution emerges, regardless of the underlying microstructure of the ITO. This underscores the efficacy of utilizing an amorphous NiO_*x*_ buffer layer as a surface modifier, effectively countering potential nonuniformities, and promoting uniform electronic response in the films, particularly in the case of *poly-ITO-μm grain* films.

Following KCl-treated NiO_*x*_, 2PACz-SAMs were deposited on ITO/NiO_*x*_ substrates (Figure 2b, right column). Figures S12 and S13 and text within the SI further demonstrate that remaining KCl crystals on the NiO_*x*_ surface after the KCl treatment are washed away after the 2PACz-SAMs deposition. With the incorporation of 2PACz-SAMs, the uniform CPD was preserved for all of the studied ITOs/NiO_*x*_, but the lower CPD values (as compared to the case of ITO/NiO_*x*_ alone) serve as evidence of the presence of 2PACz on the surface, also indicating a WF increase. The WF values were later confirmed by UPS (Figure 4). We argue that the enhancement in CPD uniformity achieved by introducing a NiO_*x*_ layer on top of ITO before 2PACz anchoring is the outcome of a synergistic interplay among its amorphous nature (i.e., nonpreferred crystal orientation) and increased hydroxyl group concentration, rather than their individual contributions as previously reported.^40,42,49^

### Colocalization KPFM and EBSD Mapping: Correlating ITO Grain Orientation with Work Function

**Figure 3:**
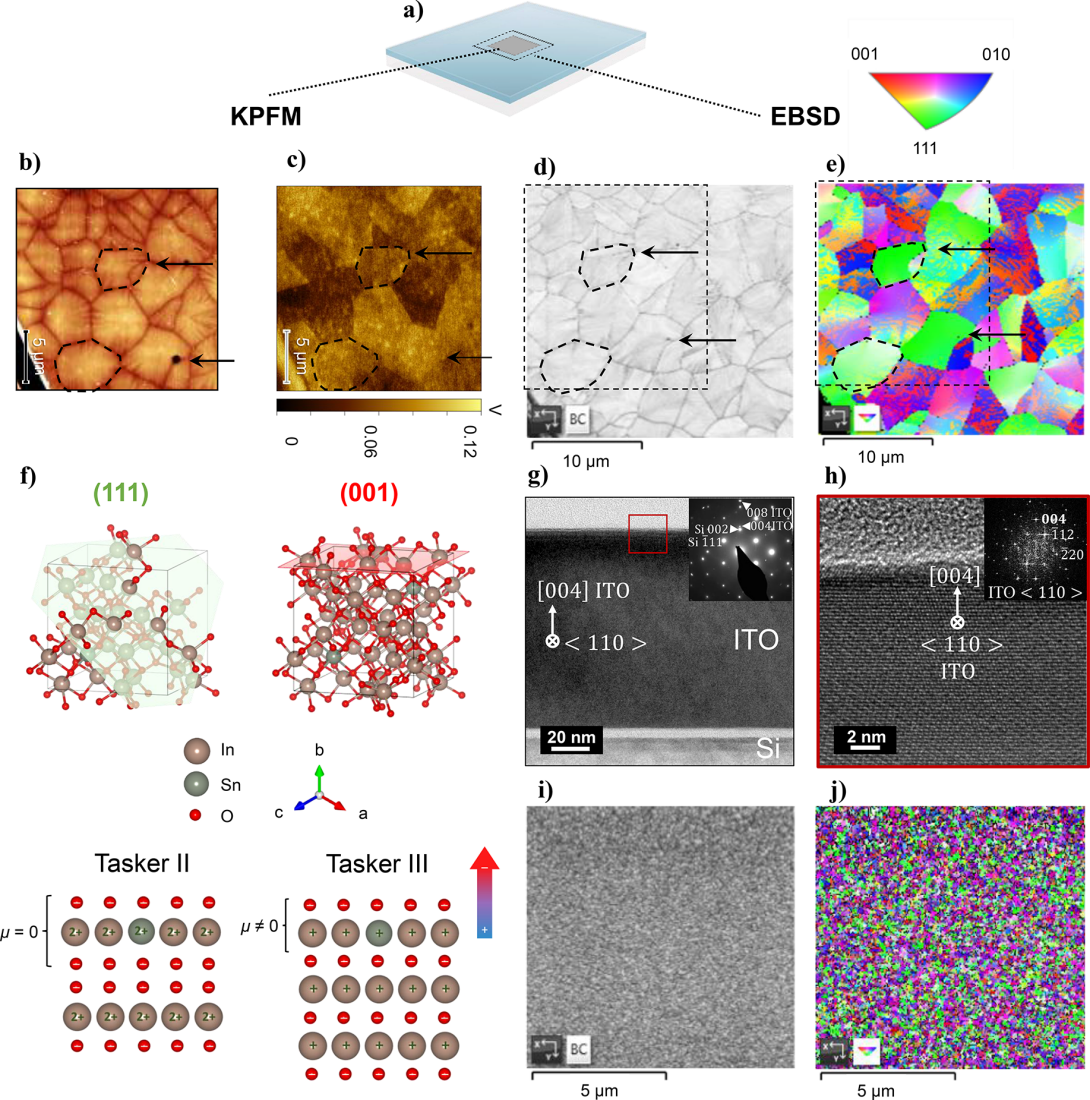
Grain orientation influence on the local WF. (a) Schematic representation of the point of interest (POI) on the sample; (b) topography and (c) CPD mapping from KPFM measurements; (d) SEM image and (e) EBSD mapping of the poly-ITO-μm-grain ITO film; (f) representation of (001) and (111) plane orientations for ITO and analogous Tasker surfaces; (g) cross-sectional TEM image and SADP (inset) of the poly-ITO-μm-grain film and (h) HR-TEM and the corresponding fast-Fourier-transform (FFT) (inset) image of the top surface showing preferential surface termination in 4×(001) orientation; (i) SEM image and (j) EBSD mapping of the poly-ITO-nm grain ITO film (The black dashed square in d-e represents the area on which both KPFM and EBSD were performed. Arrows and dash-framed grains serve as guiding marks.).

The KPFM findings reveal a notable correspondence between the CPD domains and the respective ITO grains in the *poly-ITO-μm-grain* films. To ascertain whether the identified domains originate from distinct crystalline orientations, a combined approach employing EBSD mapping and KPFM measurements is adopted for simultaneous topographical, electronic, and microstructural imaging of the ITO surface on the same point of interest (POI). This is schematically represented in Figure 3a (details on the protocol procedure^50^ in the SI).

It is well-established that crystal orientations can lead to diverse atomic arrangements on a material’s surface, influencing the electronic structure and impacting the WF. It has been proposed that generally, closely packed planes (high atomic density) display higher WF compared to loosely packed planes (low atomic density).^51^ From reported surface densities based on density functional theory calculations for In_2_O_3_ (100) > (110) > (111), it is speculated that ITO (100) planes possess higher WF.^52^ Another theory that may elucidate the phenomenon of crystalline orientation’s influence on local WF variation is the surface polarity concept, introduced by Tasker.^53^ While the initial observations were for In_2_O_3_,^52,54,55^ this concept can be extended to ITO. In detail, the (111) plane is a Tasker II type of surface without surface dipole perpendicular to the surface normal. On the other hand, the (100) plane corresponds to a Tasker III type of surface, characterized by alternating charged planes that lead to a dipolar moment on the uppermost surface layer pointing away from the surface in the normal direction. Consequently, this induced surface dipole makes the removal of an electron more challenging, resulting in an increased WF^14^ (Figure 3f).

Topography and CPD maps obtained from KPFM for *poly-ITO-μm-grain* films are presented in Figure 3b-c. Using the dash-framed grains and arrows as guiding marks, we note that the indicated grains exhibit a lighter coloration, implying a higher applied CPD and thus a lower WF. The respective grains in the EBSD map (out-of-plane, *z*-direction) are colored green, indicating (111) orientation (Figure 3d-e, S8). Conversely, the darker-colored grains identified on the CPD map, reflecting a low applied CPD, thus a high WF, align with the red/blue colored grains on the EBSD map, which correspond to the (001) family of planes. The preferred surface termination of a single grain along 4 × (001) was also confirmed with high-resolution transmission electron microscopy (HR-TEM) and selected area diffraction pattern (SADP) as presented in Figure 3g-h.

Furthermore, we conducted EBSD on *poly-ITO-nm-grain* films, as shown in Figure 3i-j and Figure S9. The distinct crystal orientations of the nanometer-scale grains are visible, suggesting that local WF variations induced by the grain’s orientation could also be expected. However, due to the nanometer-scale of the grains, the spatial resolution of the KPFM tip was not sufficient to detect such nanoscale variation. Despite this, it is suggested that local WF variations, even at the nanoscale, are expected on any polycrystalline sample.^51,55^ The presence of significant energetic variations across a material can lead to unwanted effects on a device level, such as an uneven charge distribution, altered electronic transport properties, and even limitations in device efficiency and performance as previously reported.^32,38,43^

**Figure 4:**
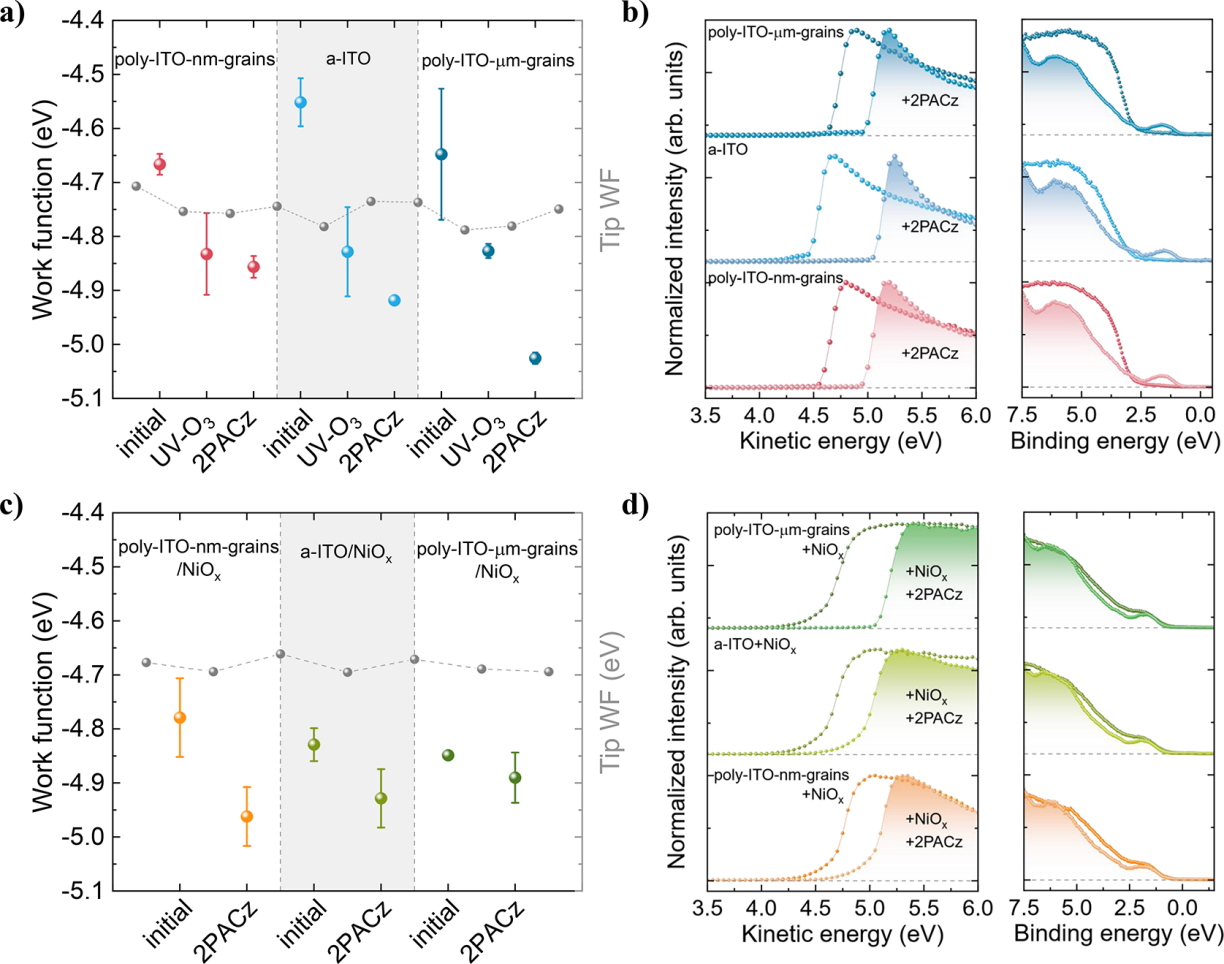
KPFM WF values (a, c), UPS secondary electron cutoff (SECO) and valence band region (b, d) for ITO with and without 2PACz (a, b) and ITO/NiO_*x*_ with and without 2PACz (c, d). (The tip WF is presented in (a, c) for reference.)

To verify the WF values determined by KPFM and to extract the valence band maximum (VBM) and highest occupied molecular orbital (HOMO) levels, UPS measurements were conducted. Figure 4a showcases the WF values determined by KPFM for the studied ITOs, in their initial state (right upon solvent cleaning procedure), after UV–O_3_ treatment and subsequent 2PACz-SAMs deposition. The large error bar for the *poly-ITO-μm-grain* film could be ascribed to the presence of the distinct crystalline grain orientations. However, the overall WF of all bare ITOs is found within the same range, and a systematic increase in the WF after UV–O_3_ treatment and upon 2PACz-SAMs anchoring is evident across all studied ITO films. This trend is expected, as any form of surface treatment inherently alters the surface potential and thereby directly impacts the WF.^51^

UPS results complement these findings, indicating a pronounced shift of the secondary electron cutoff (SECO) toward lower binding energies. This shift strongly implies a substantial WF increase upon the anchoring of 2PACz-SAMs, a phenomenon consistent across all of the examined cases. In addition, the VBM region spectra show a significant modification after 2PACz-SAMs deposition. Specifically, the characteristic sharp, linear-like form typical of TCOs–attributed to localized electronic states–changes to a hump-like HOMO edge, characteristic of organic molecules with delocalized π-electrons^56^ (Figure 4b). The presence of 2PACz-SAMs on the surface was further confirmed via X-ray photoelectron spectroscopy (XPS) analysis (Figures S10–S11).

As observed in Figure 2b (left column), the introduction of an amorphous NiO_*x*_ layer appears to mitigate surface potential disparities arising from the distinct microstructures of the ITO films. Measuring the average WF values by KPFM and UPS, only a slight variation in the WF is observed for the different ITOs after NiO_*x*_ and NiO_*x*_/2PACz-SAMs deposition (Figure 4c-d).

**Figure 5:**
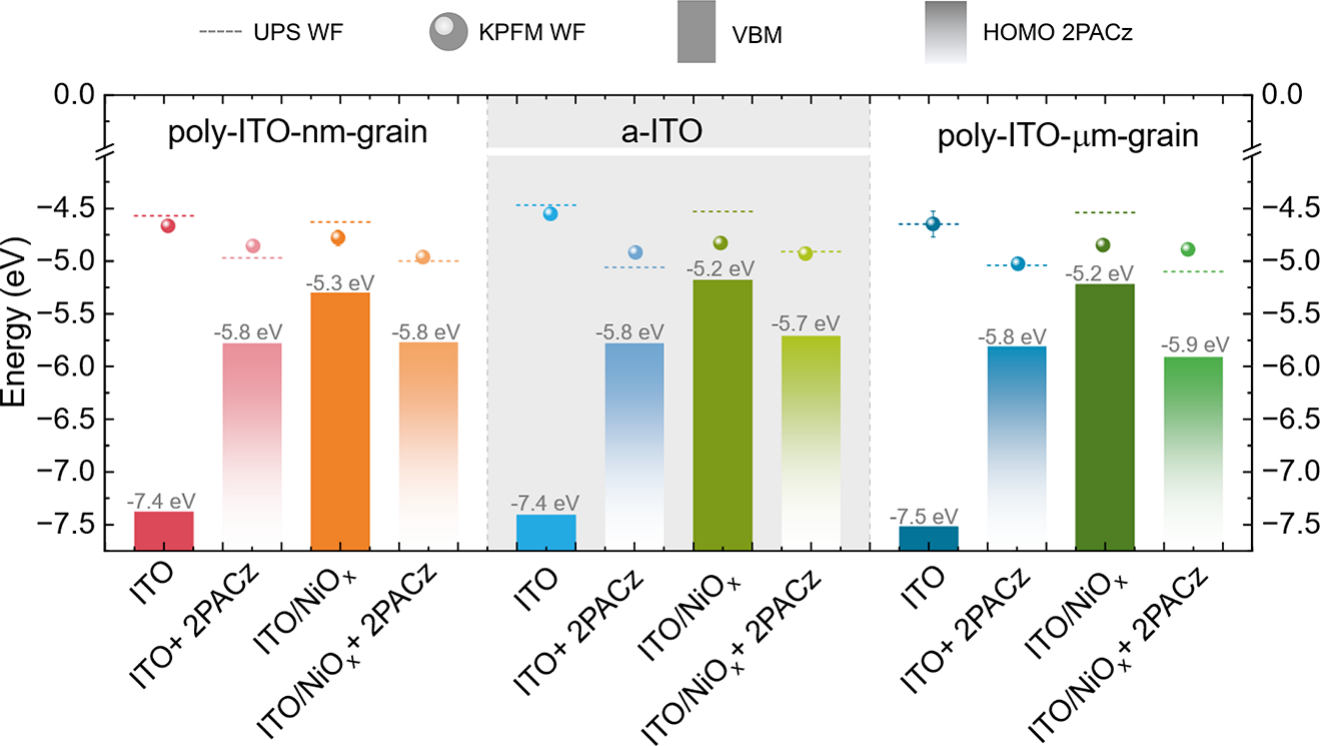
Overview of UPS and KPFM data and reconstructed energy level diagram for the distinct stacks studied (Spheres and dashed lines represent work function values determined by KPFM and UPS, respectively; gray numbers refer to ionization potential, *I_p_*, which corresponds to VBM or HOMO values for TCOs and the TCO/2PACz stack, respectively.).

The reconstructed energy level diagram represented in Figure 5 shows the values of the work function determined by KPFM (sphere) and UPS (dashed line) and ionization potential, *I*_*p*_ (numerical values in gray font). All values are listed in Table S2. An overall good correlation between two independent techniques (each operating at different conditions, i.e., UPS at ultrahigh vacuum and KPFM at ambient air) and a consistent trend in the WF increase upon ITO surface modification can be observed (for a detailed explanation, check Figures S12 and S13).

As expected, all three ITO films have a pronounced n-type character as their Fermi level is far from VBM. Modified ITOs, either with 2PACz-SAMs or NiO_*x*_ only, or a combination NiO_*x*_/2PACz-SAMs shifted the Fermi level closer to the ionization potential level (HOMO or VBM, respectively), indicating the p-type characteristics and enhanced hole selectivity. It is worth highlighting that the values of the WF and VBM for ITO/NiO_*x*_ agree with previously reported values for ALD deposited NiO_*x*_ (4.6–4.7 and 5.3 eV, respectively).^32^ Upon depositing 2PACz-SAMs, the WF is further increased, due to the molecular dipole moment that 2PACz-SAMs introduce,^47^ contributing to the surface energy term.^51^ Notably, the WF values for the ITO/2PACz-SAMs and ITO/NiO_*x*_/2PACz-SAMs cases are quite similar. Going back to the KPFM mapping, we suggest that the main advantage of NiO_*x*_ in addition to its hole transport properties is its amorphous nature, minimizing pinhole formation and ensuring a uniform WF distribution due to the lack of preferential grain orientation. We therefore argue that the use of an amorphous metal oxide with adequate WF for hole (or electron) extraction or an amorphous TCO buffer layer ensures an enhanced coverage of the 2PACz-SAMs and, with it, a uniform WF distribution. This combination holds the potential to improve energy level alignment and charge extraction on a device scale, thereby enhancing the overall device performance, reproducibility, and stability.

In summary, we studied the impact of the ITO microstructure on the electronic properties of hole-selective transport layers NiO_*x*_ and 2PACz-SAMs. Three different types of ITO thin-films morphology and microstructure were characterized. Correlated KPFM and EBSD mapping revealed that polycrystalline ITO films present a nonuniform distribution of the surface potential, subsequently impacting WF uniformity. The application of 2PACz-SAMs was not sufficient to overcome the lateral inhomogeneity in the WF inherent to the polycrystalline ITO films. However, we found that this challenge can be successfully addressed by employing either an amorphous TCO or an amorphous NiO_*x*_ buffer layer, where there is no preferential grain orientation, and low surface roughness is ensured. In the context of applying TCOs and PACz-SAMs in solar cells, e.g., in perovskite/silicon tandems, polycrystalline TCOs offer attractive properties, such as high mobility and minimum parasitic absorption losses from the visible to the near-infrared wavelengths. To overcome the nonhomogenous WF distribution upon hole transport SAMs anchoring on polycrystalline TCOs, an amorphous buffer layer (or surface modification) is recommended to ensure a uniform potential distribution and minimum nonradiative losses at the perovskite/HTL interface, ultimately improving either the device performance or reducing device-to-device variations.

## Supporting Information Available

Detailed description of used materials, applied experimental methods and procedures; elaborated XRD data, annealing series data, Tauc plot, KPFM and UPS WF values in tabular format, KPFM maps of ITO/NiO_*x*_ KCl treated surface, XPS survey spectra, EBSD analysis (PDF)
