# Supplementary material for: Impact of the TCO Microstructure on the Electronic Properties of Carbazole-Based Self-Assembled Monolayers
Source: ACS Mater Lett. 2023 Dec 26;6(2):366–74. doi: 10.1021/acsmaterialslett.3c01166 (PMC10848288; doi:10.1021/acsmaterialslett.3c01166)
Supplement: Supplementary file 1 — tz3c01166_si_002.pdf [file tz3c01166_si_002.pdf]

Supporting information

Impact of the TCO Microstructure on the Electronic Properties of Carbazole-based Self-Assembled Monolayers

Suzana Kralj [1a], Pia Dally [2], Pantelis Bampoulis [1b], Badri Vishal [2], Stefaan De Wolf [2], Monica Morales-Masis [1a]**

[1a] S. Kralj, M. Morales-Masis

MESA+ Institute for Nanotechnology, University of Twente, Enschede 7500 AE, The Netherlands

[1b] P. Bampoulis

Physics of Interfaces and Nanomaterials, MESA+ Institute for Nanotechnology, University of Twente, Enschede 7500 AE, The Netherlands

[2] P. Dally, B. Vishal, S. De Wolf

KAUST Solar Center (KSC), Physical Sciences and Engineering Division (PSE), King Abdullah University of Science and Technology (KAUST), Thuwal 23955-6900, Kingdom of Saudi Arabia

Corresponding Author

* S. Kralj (s.k.kralj@utwente.nl) * M. Morales-Masis (m.moralesmasis@utwente.nl)

Methods Section

Materials

ITO commercial substrates were purchased from *Ossila Ltd*, the PLD ITO target from *Toshima Materials Co., Ltd* with SnO_2/In_2O_3 of 10/90 wt%. 2-(9H-Carbazol-9-yl)ethyl)phosphonic acid (2PACz) >98.0% from *Tokyo Chemical Industry Co., Ltd*, Anhydrous Ethanol (max. 0.003% H_2O, ≥99.8%) from *VWR Chemicals*, potassium chloride (KCl), 99.0% from *ThermoFisher*.

Methods

Full area glass/ITO substrates (15 mm x 20 mm) were cleaned through sonication in Hellmanex® solution (2 vol.% in deionized water), deionized water, acetone and 2-propanol consecutively for 10 minutes each and dried using nitrogen gun. SAMs solution was prepared following the procedure reported by Al-Ashouri *et al*.[1] In short, 1 mg/mL 2PACz was dissolved in anhydrous ethanol. Prior to spin-coating, glass/ITO substrates were treated 15 min in UV-O_3 to activate the surface. 100 μL 2PACz solution was then spin-coated statically on samples with freshly activated surface. After approximately 5 s resting of 2PACz solution, the spin-coating program was started. Used settings were 5000 rpm for 30 s. Upon that, an annealing step was performed at 100 ˚C for 10 minutes to allow phosphonic acid anchoring group to bind to the substrate. To remove any excess unbound molecules, a washing step was introduced. 2 x 100 μL of anhydrous ethanol was spin-coated dynamically using the same program. As a final step, 5 min drying at 100 ˚C was done.

Pulsed Laser Deposition of ITO thin films. Indium tin oxide (ITO) films were prepared by Pulsed Laser Deposition (PLD) following a previously reported procedure[2]. Films were deposited on glass substrates (*ECOGlass SCHOTT*) which were cleaned prior to deposition in ultrasonic bath for 5 minutes in acetone and isopropanol and rinsed in deionised water,

respectively. All depositions were performed in a wafer-scale PLD system designed by *Solmates BV (now LAM research)*. The SnO_2/In_2O_3 (90/10 wt%) target was ablated using a COMPexpro (*COHERENT*) KrF excimer laser (λ = 248 nm) with a fluence of 2.6 J/cm^2 and frequency of 10 Hz. Deposition pressure was controlled to 0.02 mbar (50/50% Ar/O_2). Number of scans (pulses) was adjusted to keep the thickness of as-deposited films at 100 nm. Exact thickness value was estimated by X-Ray Reflectivity (XRR).

For the polycrystalline ITO, an additional annealing step was performed on as-deposited PLD ITO films. Annealing was performed at 450 ˚C for 20 minutes under a controlled environment of 5% hydrogen in nitrogen gas atmosphere.

Deposition of NiO_x hole transport layer. NiO_x layers were deposited by radio frequency (RF, 13.56 MHz) magnetron sputtering from a NiO stoichiometric target (99.95% purity) at room temperature using *Angstrom Engineering EvoVac* system at KAUST Solar Centre. The base pressure for deposition was <5x10^{-7} Torr. Deposition was performed under 3 mTorr with 20 sccm Argon gas flow. Before each deposition, to remove the contamination layer on the surface of the target (if any), the target was pre-sputtered for 15 min. For homogenous deposition, substrate rotation was provided. Thickness was controlled to ~14 nm (measured by ellipsometry on co-deposited silicon substrate).

KCl surface passivation. KCl was dissolved in deionized water (3.5 mg/mL). Solution was put on vortex mixer for 30 minutes. 120 µL solution was spin-coated on NiOx film followed by 10 minutes annealing at 130 ˚C. For case of further processing 2PACz layer, no additional surface activation step was performed and the spin-coating setting are the same as for ITO substrates.

Kelvin Probe Force Microscopy (KPFM). Amplitude modulated Kelvin Probe Force Microscopy (AM-KPFM) was measured by Bruker Dimension Icon AFM using SCM-PIT-v2 tip in dual pass mode. All measurements were performed under ambient conditions. Calibration

of the tip was executed using reference samples: i) PFKPFM-SMPL (Kelvin Probe Sample Aluminium and Gold on Silicon) at the beginning of measurement, before changing the sample and at the end of measurements to track stability of probe and ii) freshly cleaved highly ordered pyrolytic graphite (HOPG) surface before each measurement to accurately calibrate probe. Area of 10x10 μm^2 was scanned in all cases. The mapping was performed with scanning speed of 0.75 Hz. Scanning resolution was 512x512 lines in fast and slow-scan axis, respectively. For the second pass, z-height of 10 nm was used. For each condition minimum of 3 (or more) spots on the sample were measured to ensure enough data set for statistical analysis. The work function was calculated using formula[3, 4]:

$$CPD = \frac{\phi_{Probe} - \phi_{Sample}}{|e|}$$

Which is modified for probe calibration to:

$$\phi_{Probe} = \phi_{Reference} + CPD \cdot |e|$$

while for sample work function calculation to:

$$\phi_{Sample} = \phi_{Probe} - CPD \cdot |e|$$

where *CPD* equals to applied average contact potential difference, Φ_{Probe} represents the work function of probe (SCM-PIT-v2), $\Phi_{Refernce}$ represents the work function of reference samples (theoretical work function values used for reference samples were -5.1 eV and -4.5 eV for Au and HOPG[3], respectively) and $\Phi_{Refernce}$ represents the work function of the sample.

Ultraviolet-Visible-Near Infrared Spectrophotometry (UV-Vis-NIR). Optical properties of ITO thin-films were measured with a *PerkinElmer Lambda 950S* UV-Vis-NIR spectrophotometer with integrating sphere. Transmittance (T) and reflectance (R) were measured while absorptance (A) was calculated as: A = 100 – T – R.

Hall effect measurements. Electrical properties of the ITO films were measured using a Hall effect *ezHEMS NanoMagnetics Instruments* set-up in the Van der Pauw configuration at room temperature and with a magnetic field of 0.9642 T. Samples were cut into 10 mm x 10 mm pieces, input thickness value was 100 nm and applied source current, I = 100 μA.

Corelation KPFM and EBSD measurement. The protocol procedure by Maryon et al. was followed[4]. Initially, the sample's surface was mechanically scratched to establish a reference mark for subsequent point-of-interest (POI) identification. Subsequently, solution cleaning procedure was performed to remove any residual particles from the surface. The reference mark was firstly found with optical microscope and it was marked as a point of origin. KPFM measurements were conducted on randomly selected area close to proximity of reference line and its coordinates (X, Y) were stored for later co-localized measurement. The parameters used are the same as for standard KPFM measurements. EBSD was performed after KPFM measurements to prevent deposition of unwanted carbon coating on the sample surface due to exposure to the electron beam. Sample was mounted on a SEM holder using silver paste to ensure good contact and conductivity of the top surface to prevent static electric charge accumulation. The sample-carrying stage was tilted to 70˚ to enhance the diffracted signal. The scanned surface was adjusted to be within the same region as mapped with KPFM. Measurements were conducted with the 15 kV electron acceleration voltage and at working distance < 15 mm.

Transmission electron microscopy (TEM) and focused ion beam (FIB). For the Transmission Electron Microscopy (TEM)-based study, a cross-sectional electron-transparent lamella was meticulously prepared using a focused ion beam (FIB) within the Scanning Electron Microscope (SEM-FIB Helios G5 DualBeam, FEI), equipped with an EasyLift nanomanipulator and a Gallium (Ga) ion source. To protect the region of interest during FIB processing, a multi-layer protection layer were deposited. Initially, a 0.5 μm layer of both

Carbon (C) and Tungsten (W) was deposited using an electron beam (e-beam), followed by an additional 3 μm layer of W deposited by the ion beam, providing a robust protection to thin film. The ion beam milling proedure was carried out step by step with decresing beam currents (ranging from 2.4 nA to 0.025 nA, over an accelerating voltage range of 30 kV to 5 kV). This precise process allowed for the gradual cutting and thinning of the lamella down to a thickness of 60 nm while minimizing ion beam-induced damage. Furthermore, a low-current cleaning procedure (performed at voltages ranging from 5 kV to 2 kV and currents from 81 pA to 28 pA) was executed to ensure the removal of any potential contamination. Subsequent TEM-based experiments were conducted using the Cs-corrected ThermoFisher Titan 60-300 Cubed TEM microscope, operating at an acceleration voltage of 300 kV. The acquired TEM data underwent thorough processing using specialized software packages, including Gatan™ Digital Micrograph and Thermo Scientific™ Velox suites.

Structure visualization Software. *ChemDraw* molecule editor and visualization software was used to create molecule of 2PACz. *VESTA* was used to visualize plane orientations in In_2O_3. Input data file was taken from Crystallography Open Database (COD) for In_2O_3 (*CIF 2310009*)[5] and modified with replacing two Indium (In) atoms for Tin (Sn) atoms to illustrate the Sn-doping of In_2O_3.

UPS/XPS. Ultraviolet photoelectron spectroscopy (UPS) and X-ray photoelectron spectroscopy (XPS) measurements were conducted using an Omicron multi-probe Photoemission Spectroscopy (PES) system (base pressure of $5x10^{-10}$ mbar) equipped with a Sphera II EAC 125 hemispherical electron analyser and a multi-channeltron electron detector. The surface WF and valence region were studied by UPS with a vacuum ultraviolet unfiltered He(1) (21.22 eV) source (focus) (90 mA, 570 V). The samples were biased to -10 V to observe the secondary electron cut-off (SECO)[6]. The photoelectrons were collected at an angle of 80° between the

sample and analyser, with a normal electron take off angle. The constant analyser pass energy (CAE) was 5 eV for the valence band region and for the SECO.

XPS was conducted with a monochromatic Al Kα 1486.6 eV X-ray source, operating at a power of 390 W. The photoelectrons were also measured from the same spot as used for UPS. The photoelectron pass energy was fixed at 15 eV for high-resolution XPS and 50 eV for survey. Quantification of XPS spectra were conducted in CASAXPS software, integrating the peak areas using a Tougaard based background function.

The value of ionization potential, I_P was calculated from UPS data using formula[7]:

$$I_p = 21.2\ eV - (E_{SECO} - E_{VB\ or\ HOMO,UPS})$$

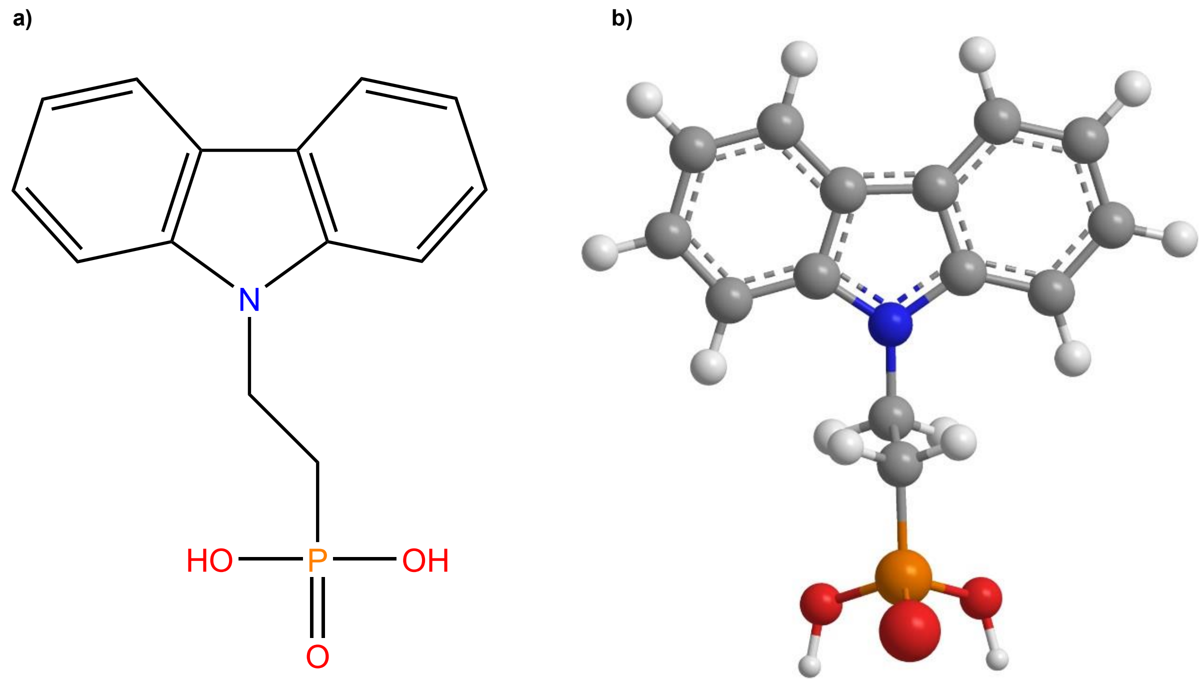

Figure S1. [2-(9H-carbazol-9-yl)ethyl] phosphonic acid (2PACz): **a)** Chemical structural formula and, **b)** Three-dimensional structure (atom coloration: carbon (C) in grey, hydrogen (H) in white, nitrogen (N) in blue, phosphorous (P) in orange, and oxygen (O) in red).

Table S1. X-Ray diffraction peak positions for polycrystalline samples

In_2O_3 reference (ICSD code: 14388)		Commercial ITO (polycrystalline, nm-size grains)	PLD ITO anneal (polycrystalline, µm-size grains)
(hkl)	2Theta	2Theta (˚)	2Theta (˚)
(211)	21.50	21.23	21.51
(222)	30.59	30.22	30.75
(400)	35.46	35.09	35.62
(411)	37.69	37.28	37.72
(422)	43.80	/	43.86
(341)	45.69	45.22	45.82
(440)	51.02	50.42	51.25
(611)	55.98	/	56.20

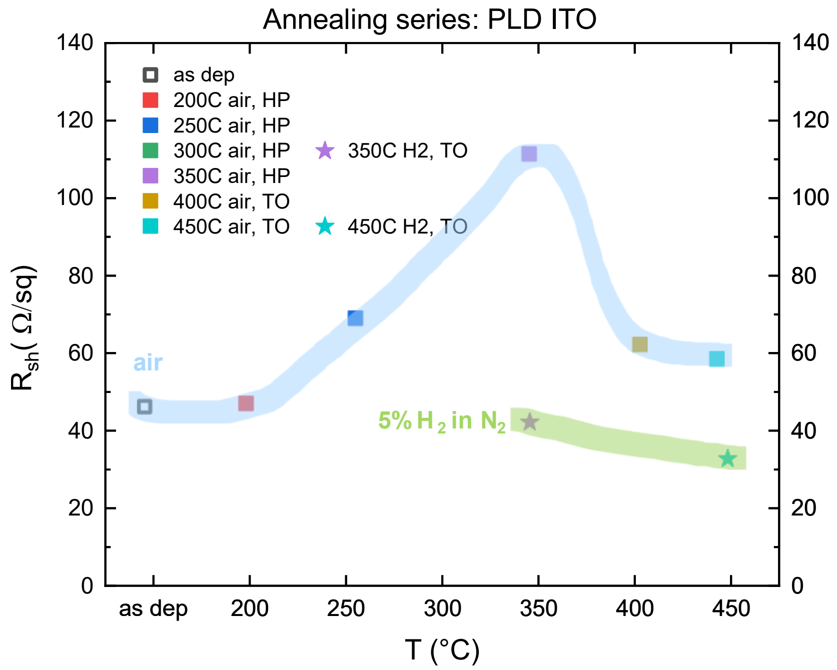

Figure S2. Post-annealing treatment of PLD ITO films at different temperatures and under different atmospheres showing sheet resistance (R_{sh}) values upon annealing. The film annealed at 450 ˚C under a 5% H_2/N_2 atmosphere exhibited the lowest R_{sh} and was consequently chosen for further investigation.

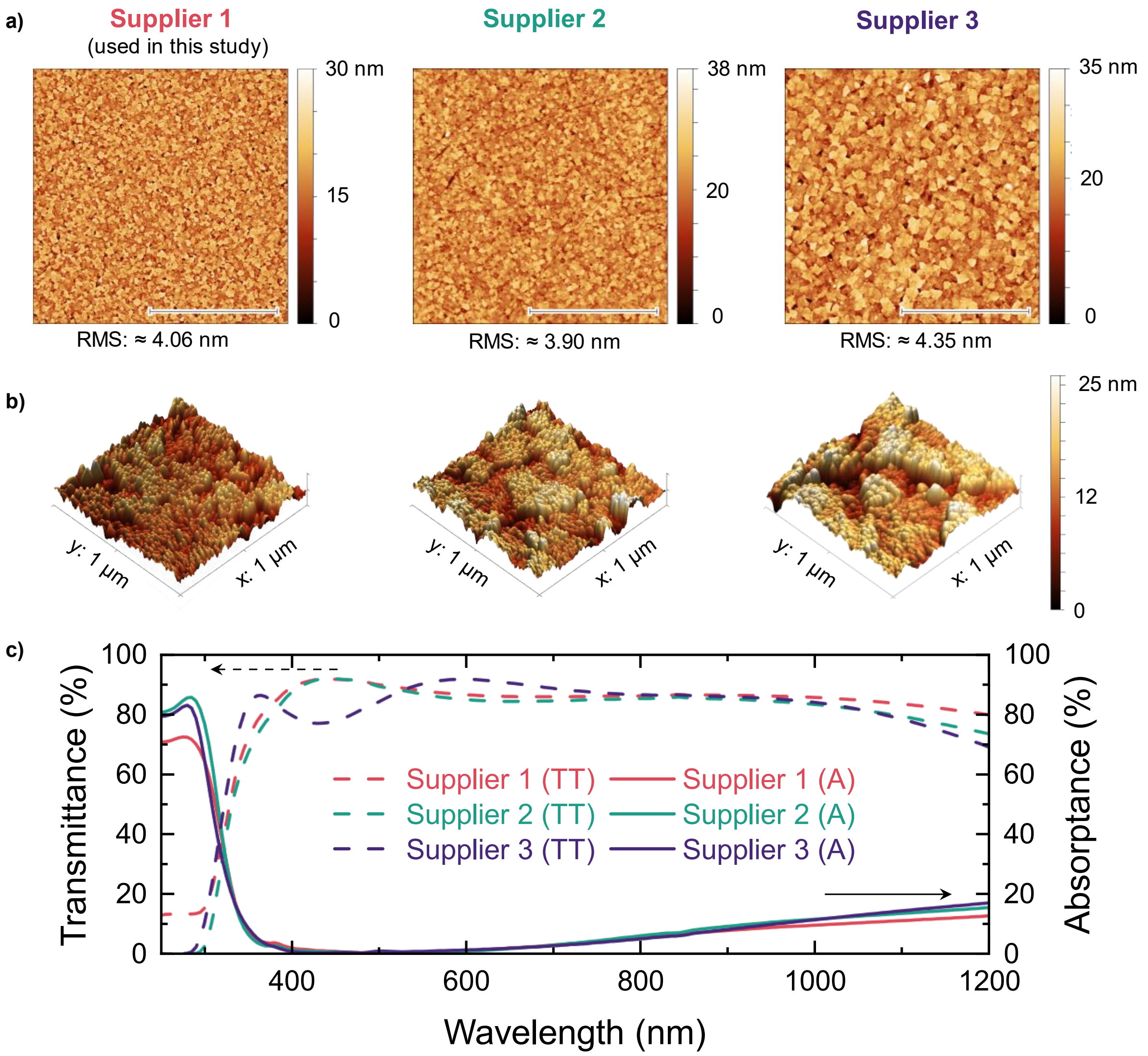

Figure S3. Commercially available ITOs from different suppliers: **(a)** AFM topography images of 10x10 μm^2 scan (the scale bar is equivalent to 5 μm); **(b)** 3D AFM topography image of 1x1 μm^2 scan; and **(c)** Optical properties: total transmittance (TT) and absorptance (A). The R_{sh} of the films is 17 Ohm/sq for supplier 1, 15 Ohm/sq for supplier 2 and 8 Ohm/sq for supplier 3.

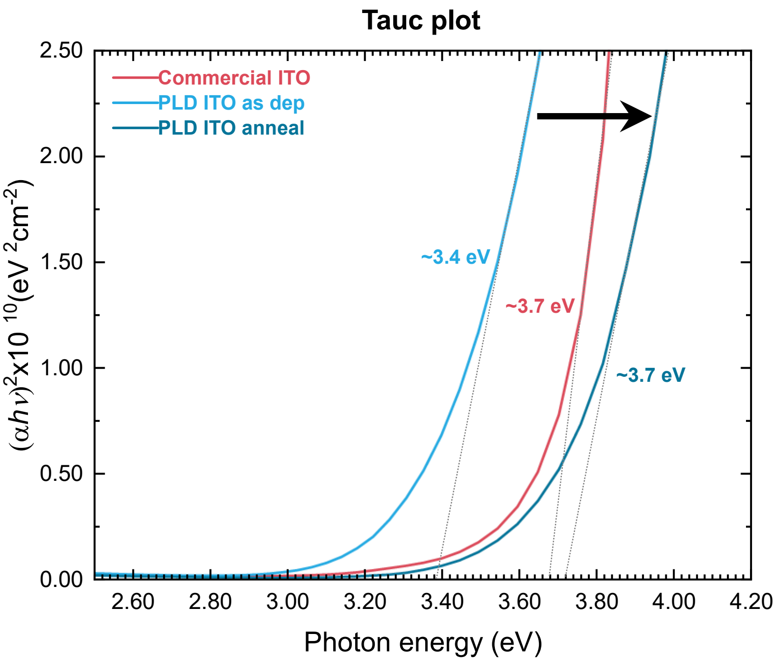

Figure S4. Tauc plot for studied ITO films with extracted band gap values

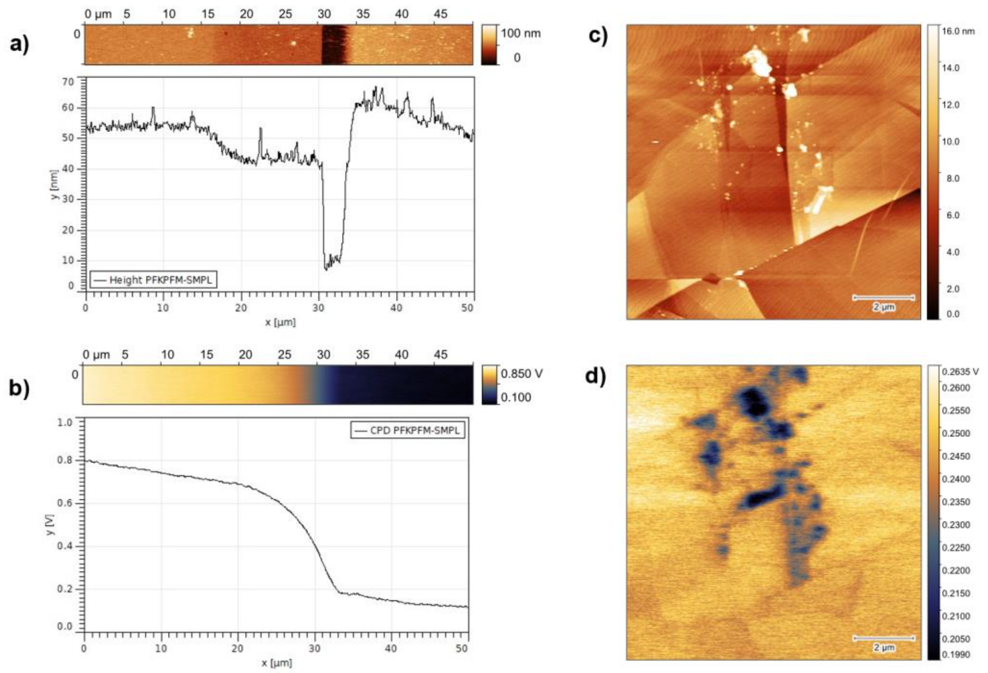

Figure S5. KPFM probe calibration (SCM-PIT v2). Topography (**a,c**) and Contact potential difference mapping (**b,d**) for PFKPFM-SML (**a,b**) and HOPG (**c,d**) reference samples.

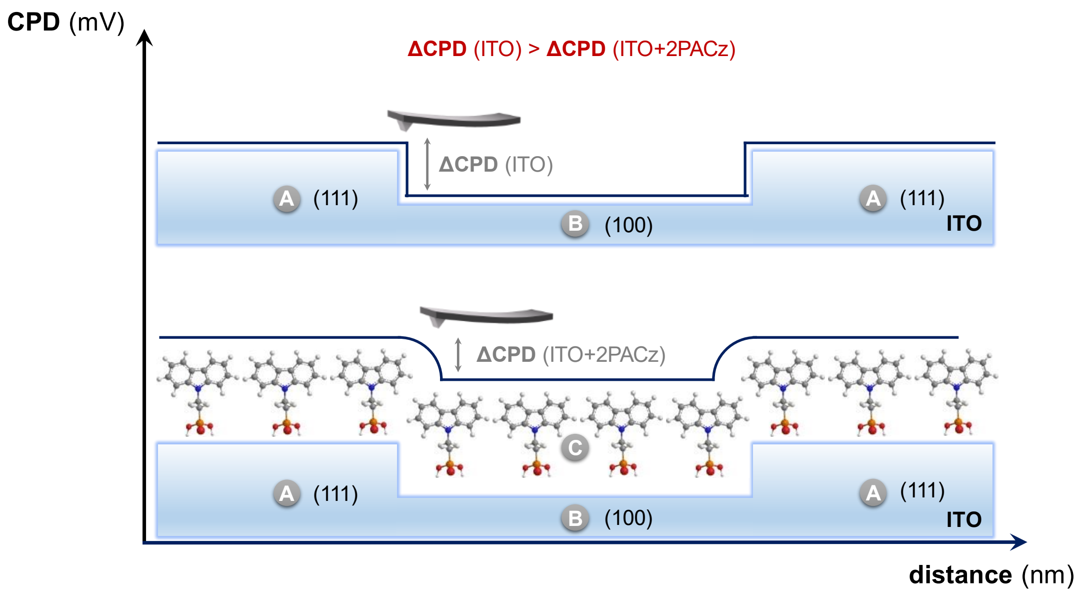

Figure S6. Schematic representation of interaction dynamics of the tip, ITO-µm-grain surface and 2PACz (not up to scale).

For the poly-ITO-µm grain films a narrowing of the CPD-distribution is observed. We hypothesized that this can be a combined effect of the UV-O_3 plasma treatment before the application of SAMs which ensures an oxygen terminated surface (and a hydroxyl-rich surface upon exposure to ambient air) and by a quenching effect, which is related to the interaction dynamics between the tip, the surface, and the 2PACz molecules (**Figure S6**). The tip interacts directly with the ITO surface, following its potential features ascribed to the different ITO grains with differing work functions (domains A and B), as presented in **Figure S6**, top. Upon the UV-O_3 treatment and the 2PACz adsorption (C in **Figure S6**, bottom) on the different ITO grains, the 2PACz interact with the domains, reducing the disparity in work function between A and B. Moreover, the AFM tip interacts mainly with material C, with a specific work function, further diminishing the underlying difference between A and B.

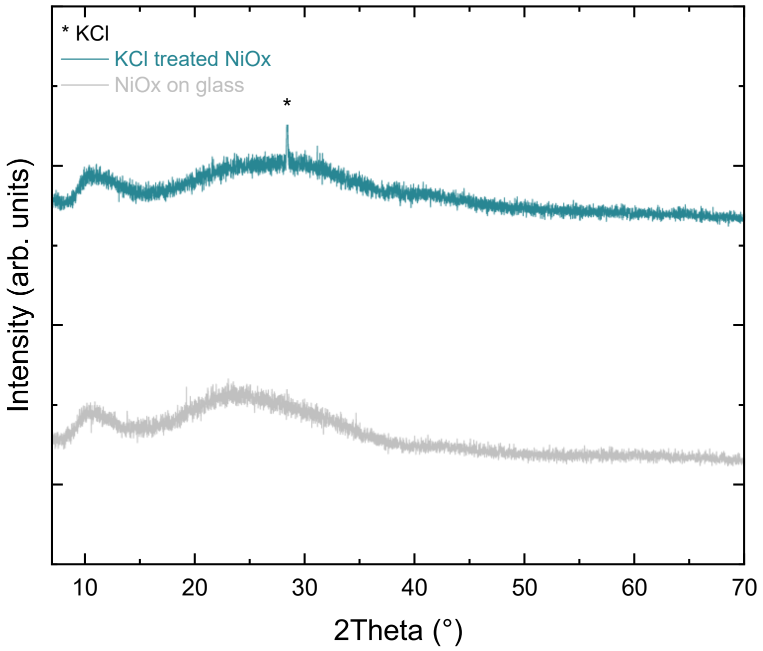

Figure S7. XRD of NiO_x (grey) and KCl treated NiO_x film (blue) indicating the presence of KCl on the surface.

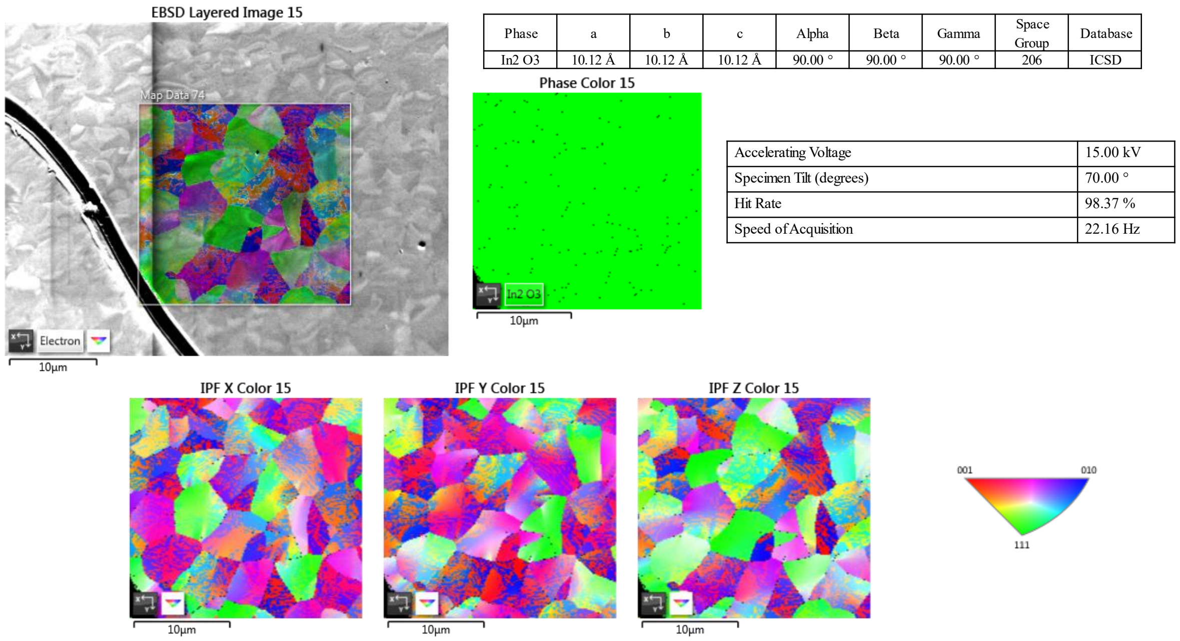

Phase	a	b	c	Alpha	Beta	Gamma	Space Group	Database
In2 O3	10.12 Å	10.12 Å	10.12 Å	90.00 °	90.00 °	90.00 °	206	ICSD

Accelerating Voltage	15.00 kV
Specimen Tilt (degrees)	70.00 °
Hit Rate	98.37 %
Speed of Acquisition	22.16 Hz

Figure S8. Comprehensive EBSD characterization of poly-ITO-μm-grain film.

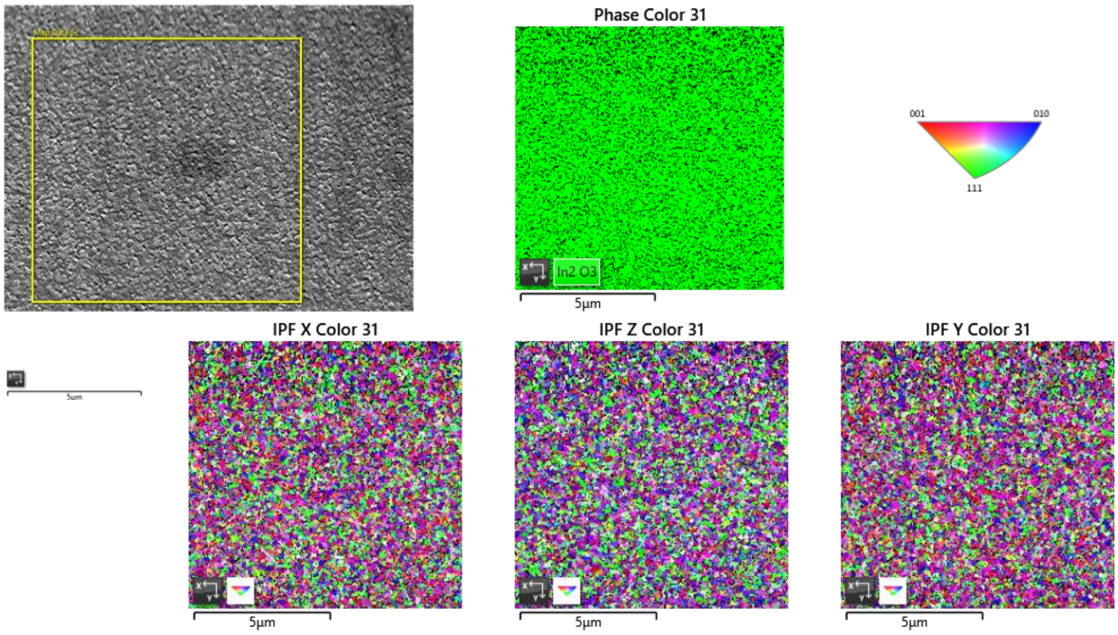

Figure S9. Comprehensive EBSD characterization of poly-ITO-nm-grains film.

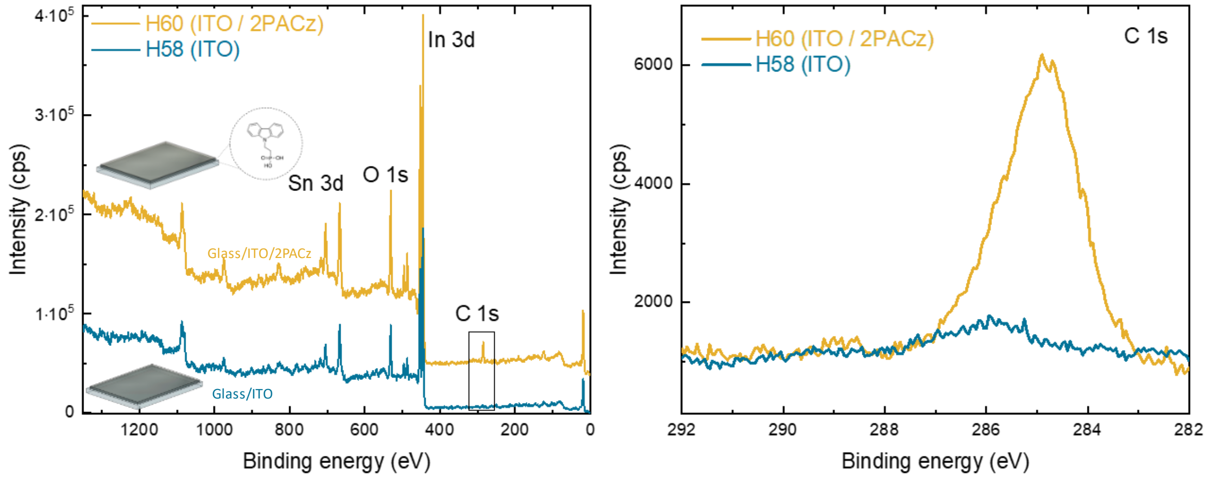

Figure S10. XPS survey spectra for ITO (blue) and ITO + 2PACz (yellow). The high resolution XPS spectra for C 1s is shown, indicating the presence of 2PACz-SAM

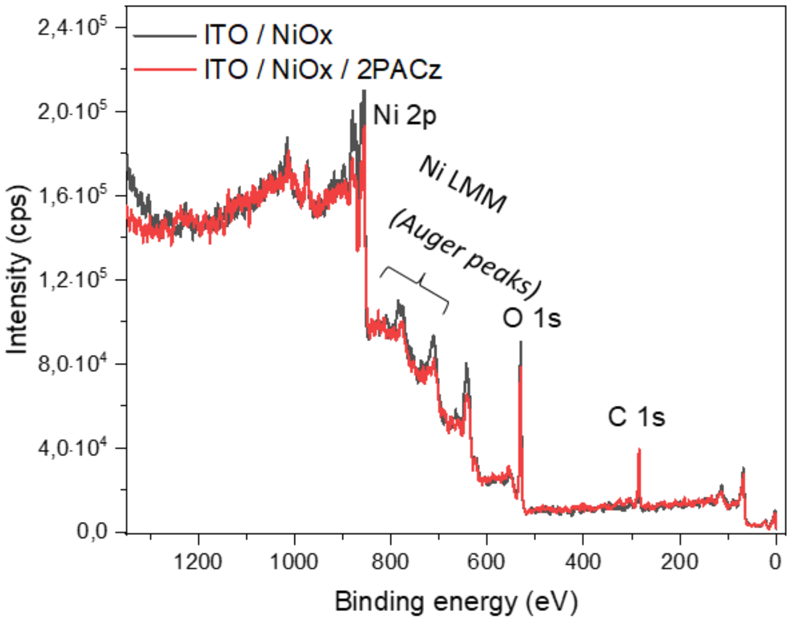

Figure S11. XPS survey spectra for ITO/NiO_x (black) and ITO/NiO_x + 2PACz (red)

Table S2. Summary of average work function values determined by UPS and KPFM

Condition	WF UPS (eV)	WF KPFM (eV)	Energy onset, $E_{VB\ or\ HOMO,\ UPS}$ (eV)	Ionization potential, I_p (eV)
Commercial ITO	-4.57	-4.66	2.81	-7.38
Commercial ITO UV-O_3		-4.83		
Commercial ITO + 2PACz	-4.97	-4.86	0.81	-5.78
Commercial ITO / NiO_x	-4.63	-4.77	0.67	-5.30
Commercial ITO / NiO_x + 2PACz	-5.00	-4.96	0.77	-5.77
PLD ITO as dep	-4.47	-4.55	2.94	-7.41
PLD ITO as dep UV-O_3		-4.83		
PLD ITO as dep + 2PACz	-5.06	-4.92	0.72	-5.78
PLD ITO as dep / NiO_x	-4.53	-4.83	0.65	-5.18
PLD ITO as dep / NiO_x + 2PACz	-4.91	-4.93	0.80	-5.71
PLD ITO anneal	-4.65	-4.65	2.87	-7.52
PLD ITO anneal UV-O_3		-4.83		
PLD ITO anneal + 2PACz	-5.04	-5.02	0.77	-5.81
PLD ITO anneal / NiO_x	-4.54	-4.85	0.68	-5.22
PLD ITO anneal / NiO_x + 2PACz	-5.10	-4.89	0.81	-5.91

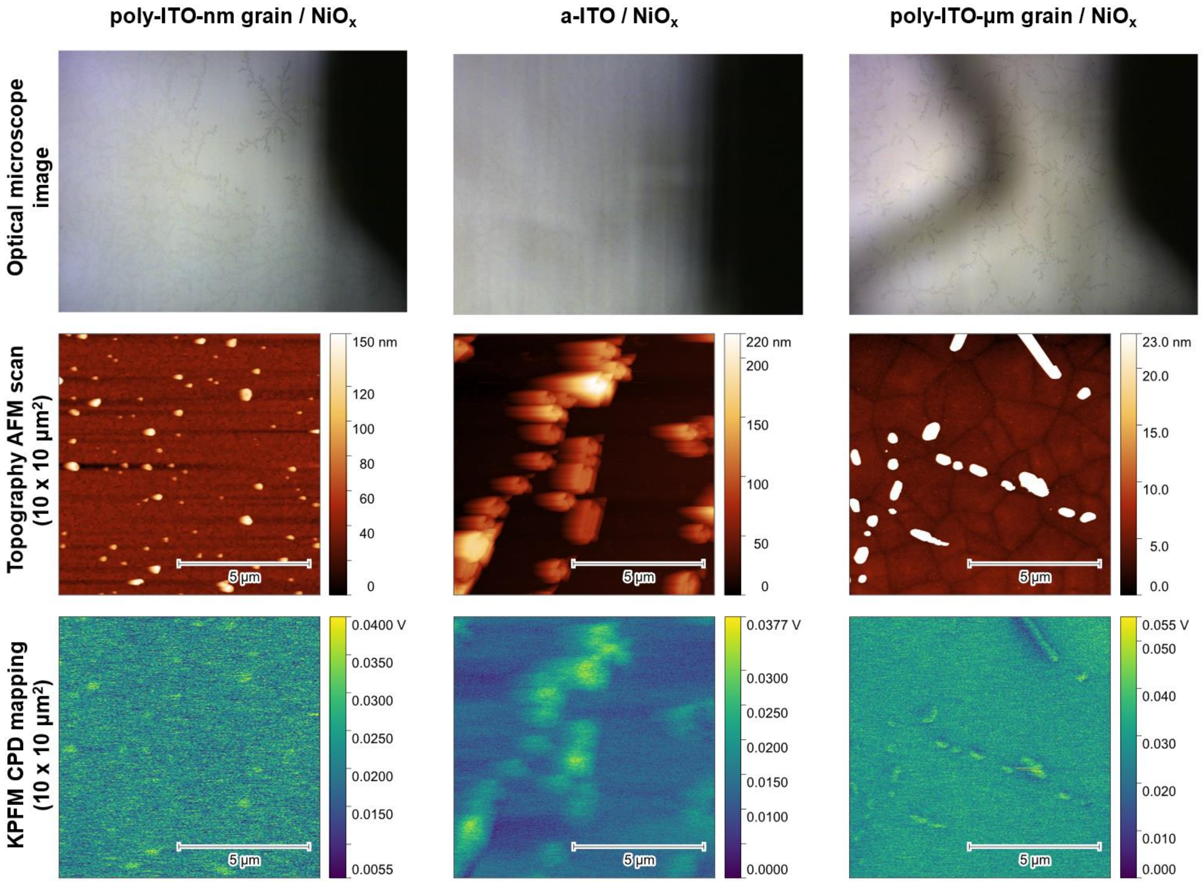

Figure S12. ITO/NiO$_x$ films after treating the NiOx with KCl solution. KCl crystals on ITOs surface captured by the optical microscope of the AFM setup (top row images); AFM topography scan (middle row images); and surface potential distribution maps showing domains of KCl crystals with higher surface potential (bottom row images). Scanned area for AFM and KPFM measurement is 10 x 10 μm^2, while the scale bar corresponds to 5 μm.

A slight discrepancy between KPFM-UPS WF values were observed for different ITO samples modified with NiO$_x$ and subjected to surface passivation treatment using a KCl solution. This can be attributed to the recrystallization of KCl on the surface of NiO$_x$ and the non-uniform distribution of KCl crystals, as previously reported by Zheng et al.[8]. The KPFM mapping presented in **Figure 2.b**, left-hand column are selected to show areas without KCl crystals, while **Figure S12** shows the areas with KCl crystals and their effect on surface potential

distribution on the ITO/NiO_x stack. To further confirm that the observed particles are indeed KCl crystals, XRD was performed. From, **Figure S7** it can be seen that as-deposited NiO_x films on glass are amorphous, while upon KCl treatment, a clear peak is observed at 28.42° which corresponds to the (200) plane of KCl. On the other side, the UPS spot size is in the order of millimetres. Therefore, these crystals were also included in averaging out the values of Fermi level causing differences in values. Nonetheless, upon depositing 2PACz, the WF values increase again as the KCl crystals are washed off by solution when processing SAMs on top and uniform CPD distribution is achieved. From **Figure *S13*** it is clear that KCl crystals are present only for the case of the KCl treated surface, with the majority of them being washed away with the ethanol washing process. Furthermore, from the surface potential distribution maps it is evident that the CPD distribution is uniform and the value of CPD is almost the same as for the case of untreated NiO_x.

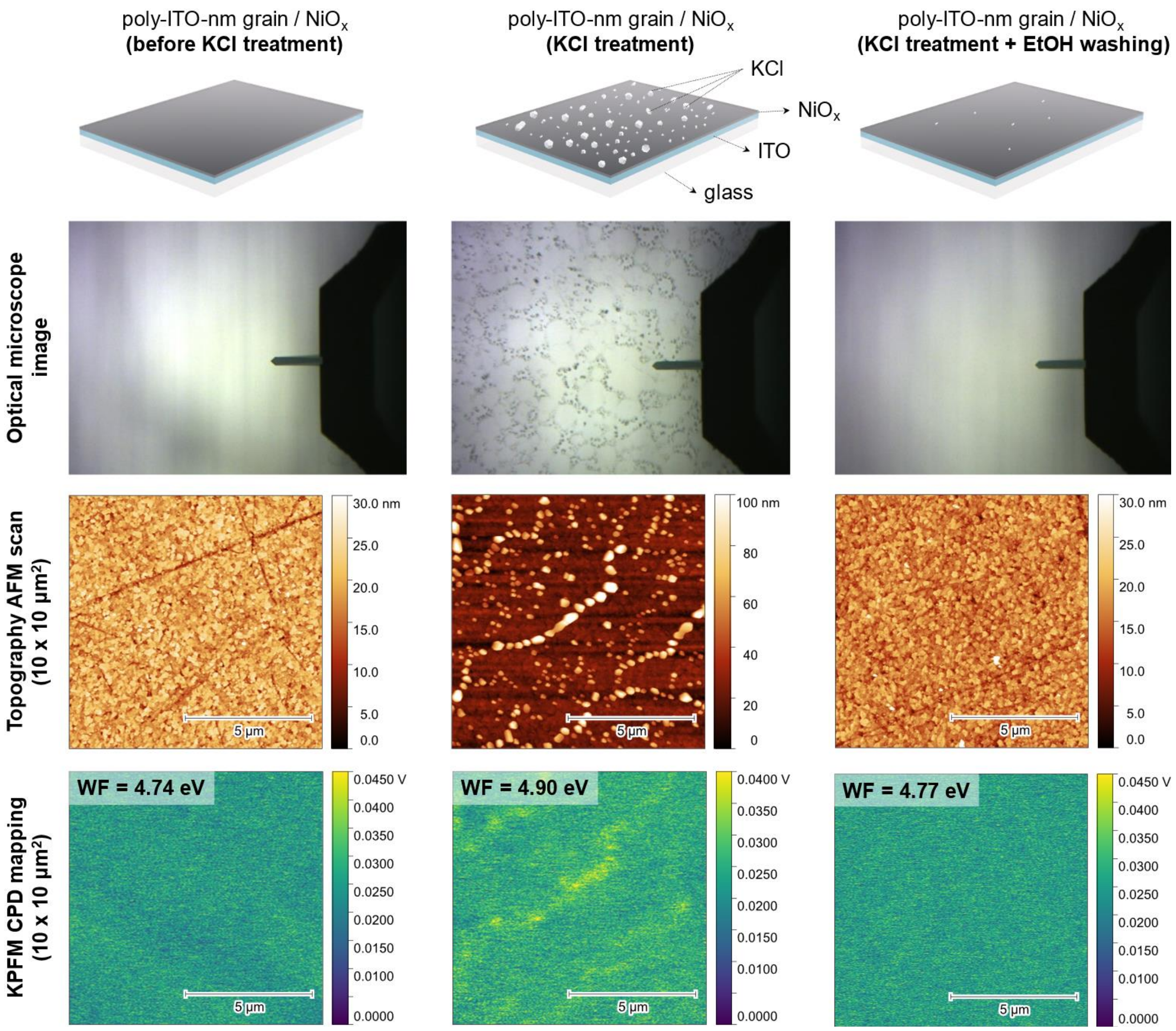

Figure S13. poly-ITO-nm grain/NiO_x films – the effect of KCl treatment and ethanol washing of KCl treated surface: schematic representation of the sample stack and surface condition (first row); optical microscope images taken by AFM setup (second row); AFM topography scan (third row); and surface potential distribution maps (fourth row). Scanned area for AFM and KPFM measurement is 10 x 10 μm^2, while the scale bar corresponds to 5 μm.

References

1. Al-Ashouri, A.; Kohnen, E.; Li, B.; Magomedov, A.; Hempel, H.; Caprioglio, P.; Marquez, J. A.; Morales Vilches, A. B.; Kasparavicius, E.; Smith, J. A.; Phung, N.; Menzel, D.; Grischek, M.; Kegelmann, L.; Skroblin, D.; Gollwitzer, C.; Malinauskas, T.; Jost, M.; Matic, G.; Rech, B.; Schlatmann, R.; Topic, M.; Korte, L.; Abate, A.; Stannowski, B.; Neher, D.; Stolterfoht, M.; Unold, T.; Getautis, V.; Albrecht, S., Monolithic perovskite/silicon tandem solar cell with >29% efficiency by enhanced hole extraction. *Science* **2020,** *370* (6522), 1300-1309, DOI: 10.1126/science.abd4016.
2. Smirnov, Y.; Repecaud, P.-A.; Tutsch, L.; Florea, I.; Zanoni, K. P. S.; Paliwal, A.; Bolink, H. J.; i Cabarrocas, P. R.; Bivour, M.; Morales-Masis, M., Wafer-scale pulsed laser deposition of ITO for solar cells: reduced damage vs. interfacial resistance. *Mater. Adv.* **2022,** *3* (8), 3469-3478, DOI: 10.1039/d1ma01225h.
3. Fernandez Garrillo, P. A.; Grevin, B.; Chevalier, N.; Borowik, L., Calibrated work function mapping by Kelvin probe force microscopy. *Rev. Sci. Instrum.* **2018,** *89* (4), 043702, DOI: 10.1063/1.5007619.
4. Maryon, O. O.; Efaw, C. M.; DelRio, F. W.; Graugnard, E.; Hurley, M. F.; Davis, P. H., Co-localizing Kelvin Probe Force Microscopy with Other Microscopies and Spectroscopies: Selected Applications in Corrosion Characterization of Alloys. *J. Vis. Exp.* **2022,** (184), e64102, DOI: 10.3791/64102.
5. Marezio, M., Refinement of the Crystal Structure of In_2O_3 at two Wavelengths. *Acta Crystallogr.* **1966,** *20* (6), 723-728, DOI: 10.1107/S0365110X66001749.
6. Schultz, T., A unified secondary electron cut-off presentation and common mistakes in photoelectron spectroscopy. *Electron. Struct.* **2022,** *4* (4), DOI: 10.1088/2516-1075/ac9ffb.
7. Whitten, J. E., Ultraviolet Photoelectron Spectroscopy: Practical Aspects and Best Practices. *Appl. Surf. Sci.* **2023,** *13*, 100384, DOI: 10.1016/j.apsadv.2023.100384.
8. Zheng, X.; Song, Z.; Chen, Z.; Bista, S. S.; Gui, P.; Shrestha, N.; Chen, C.; Li, C.; Yin, X.; Awni, R. A.; Lei, H.; Tao, C.; Ellingson, R. J.; Yan, Y.; Fang, G., Interface Modification of Sputtered NiO_x as the Hole-Transporting Layer for Efficient Inverted Planar Perovskite Solar Cells. *J. Mater. Chem. C* **2020,** *8* (6), 1972-1980, DOI: 10.1039/c9tc05759e.
